# Unusual Signal of Lymphadenopathy in Children with Nodular Sclerosing Hodgkin Lymphoma

**DOI:** 10.3390/healthcare12212180

**Published:** 2024-11-01

**Authors:** Shyam Sunder B. Venkatakrishna, Devyn C. Rigsby, Raisa Amiruddin, Mohamed M. Elsingergy, Jean Henri Nel, Suraj D. Serai, Hansel J. Otero, Savvas Andronikou

**Affiliations:** 1 Department of Radiology, Children’s Hospital of Philadelphia, Philadelphia, PA 19104, USA; 2 School of Clinical Medicine, University of Cambridge, Addenbrooke’s Hospital, Hills Rd., Cambridge CB2 0SP, UK; 3 Department of Radiology, Perelman School of Medicine, University of Pennsylvania, Philadelphia, PA 19104, USA

**Keywords:** children, magnetic resonance imaging, nodular sclerosing Hodgkin lymphoma

## Abstract

Purpose: The current guidelines for initial cross-sectional imaging in pediatric lymphomas involve computed tomography (CT) of the chest, abdomen, and pelvis. However, whole-body magnetic resonance imaging (MRI) can be favored over CT for diagnosing and staging the disease, given its lack of ionizing radiation and its higher tissue contrast. Imaging characteristics of lymphoid tissue on MRI include a high T2/short tau inversion recovery (STIR) signal. A low or intermediate signal of lymphadenopathy on T2 and STIR images is an unexpected finding, noted anecdotally in nodular sclerosing Hodgkin lymphoma. This signal may be characteristic of a histological subtype of the disease and, if confirmed, could potentially be used to avoid biopsy. In this study, we aimed to review signal characteristics of lymphadenopathy in patients with biopsy-confirmed nodular sclerosing Hodgkin lymphoma. Methods: We undertook a retrospective review of relevant MR studies of patients with nodular sclerosing Hodgkin lymphoma. Studies were reviewed by an experienced pediatric radiologist regarding lymph node signal, especially on T2/STIR. Results: Eleven children with nodular sclerosing Hodgkin lymphoma were included. Median age at the time of MRI was 14.3 (IQR: 13.9–16.1) years, and nine were boys. Five patients showed some lymphadenopathy with a low T2/STIR signal, and six showed an intermediate T2/STIR signal. Central gadolinium non-enhancement was observed in four patients. Conclusions: All eleven patients (100%) with a diagnosis of nodular sclerosing Hodgkin lymphoma showed some lymphadenopathy with a low or intermediate T2/STIR signal, and five children (45.5%) showed a frank low signal of some lymphadenopathy, a feature which may prove to be a biomarker for this histology.

## 1. Introduction

Hodgkin lymphoma is a common and treatable malignancy in children, with the nodular sclerosing subtype being its most common histological variant. The disease is assessed thorough clinical, biochemical, radiological, and histological examination. Imaging is crucial in characterizing lesions for staging, guiding biopsies for tissue diagnosis, and analyzing response to therapy. The current imaging protocols of the Children’s Oncology Group (COG) and the European Network for Pediatric Hodgkin Lymphoma (EuroNet-PHL) include radiographic and computed tomography (CT) imaging studies of the neck, chest, abdomen, and pelvis and positron emission tomography (PET) imaging for metabolic evaluation, ensuring that the radiation dose is as low as reasonably achievable. CT imaging is non-invasive and fast and has high patient acceptance. While CT is most commonly used, magnetic resonance imaging (MRI) might provide improved characterization of tumor morphology and distribution without employing radiation, and is, therefore, gaining popularity as a primary imaging modality in COG and EuroNet-PHL trials [1,2,3]. Nonetheless, the diagnosis is usually confirmed by histological examination of biopsied lymph node samples [1,4,5].

Normal lymphoid tissue is hyperintense on T2-weighted (T2w) MR images, which are often referred to as “fluid-sensitive” images. Lymphomas similarly demonstrate a hyperintense signal on T2w images due to the high water content of neoplastic tissue. Short tau inversion recovery (STIR) MR images are highly sensitive to fluid, with suppression of the signal coming from fat. Hence, similar to T2w sequences, both benign and malignant lymphoid tissue, including the thymus, show high signal intensity on STIR [1,4,5]. Lymph nodes with isointense and hypointense signal characteristics on T2w and STIR MRI sequences are not recognized as being associated with lymphomatous pathology [6]. While anecdotal descriptions of these findings are noted in the literature about biopsy-confirmed nodular sclerosing Hodgkin lymphoma [5], further studies reviewing similar observations are necessary to establish the possibility of these signal characteristics being specific to this histological subtype of the disease. This feature could prove to be a biomarker for the nodular sclerosing variant of Hodgkin lymphoma and circumvent the need for invasive biopsy for diagnosis and staging. In this study, we aimed to review signal characteristics of lymphadenopathy in biopsy-confirmed nodular sclerosing Hodgkin lymphoma.

## 2. Materials and Methods

**Figure 1:**
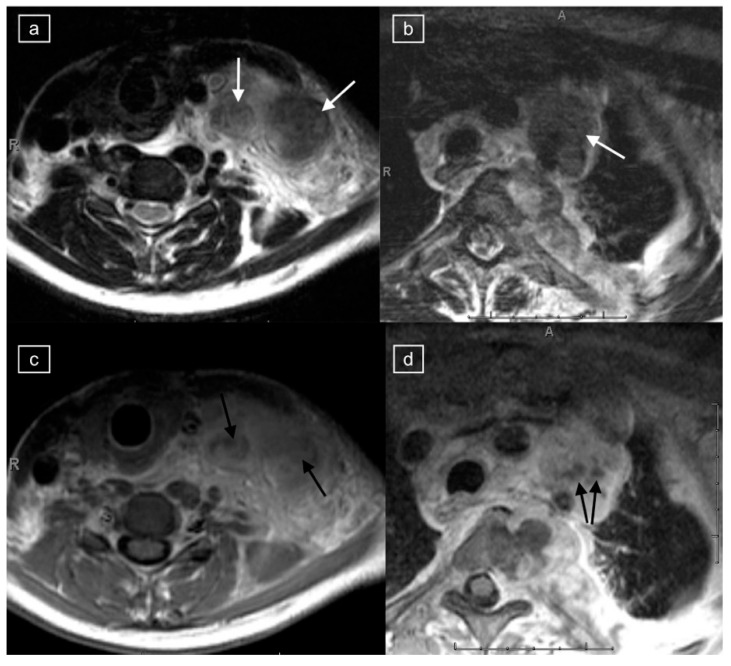
Magnetic resonance imaging (MRI) in a 20-year-old female with a histologic diagnosis of nodular sclerosing Hodgkin lymphoma. Axial T2-weighted (T2w) images of the lower cervical region (**a**) and upper mediastinum (**b**) demonstrate large left cervical and left paratracheal lymphadenopathy (white arrows) exhibiting low T2 signal. On corresponding post-gadolinium T1 imaging of the lower cervical region (**c**) and upper mediastinum (**d**), the abnormal lymph nodes show central foci of hypo-enhancement (black arrows).

**Figure 2:**
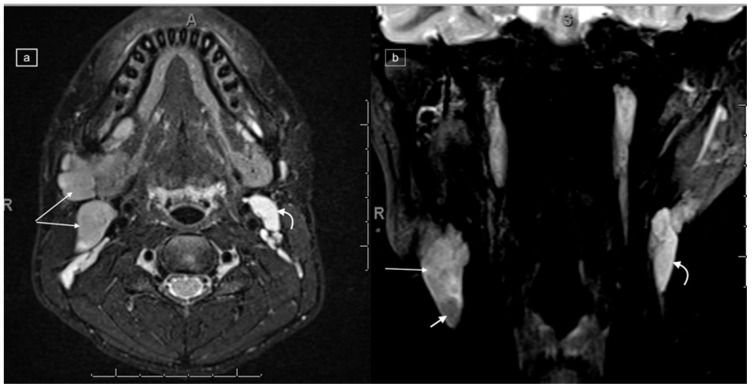
Axial (**a**) and coronal (**b**) T2w DIXON MRI in a 16-year-old male with a histologic diagnosis of nodular sclerosing Hodgkin lymphoma, demonstrating right-sided lymphadenopathy with T2 isointensity (long arrows) as compared to the normal cervical nodes on the left demonstrating T2 high-signal intensity (curved arrows). In addition, there is a focal low-signal area within the largest lymph node on the right (short arrow in (**b**)).

**Figure 3:**
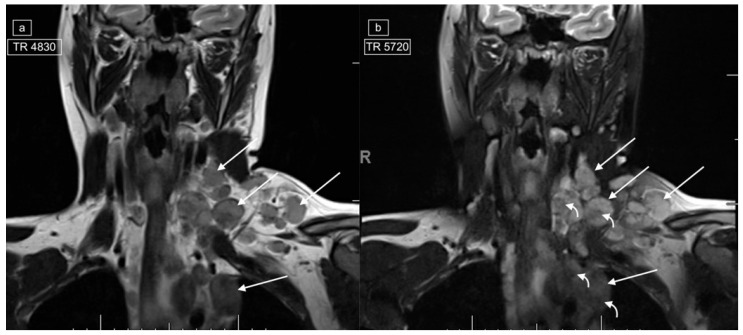
Images demonstrating technical reasons for lower T2 signal of lymphadenopathy. MRI of the neck and superior mediastinum on coronal T2 with (**a**) a repetition time (TR) of 4830 and (**b**) a TR of 5720 [fat-suppressed sequence] in a 14-year-old male with a histologic diagnosis of nodular sclerosing Hodgkin lymphoma. The left cervical and mediastinal lymphadenopathy (straight arrows) show significantly lower signal with lower TR images (**a**), but even on the higher TR images, central foci within the pathological lymph nodes demonstrate low signal (curved arrows).

**Figure 4:**
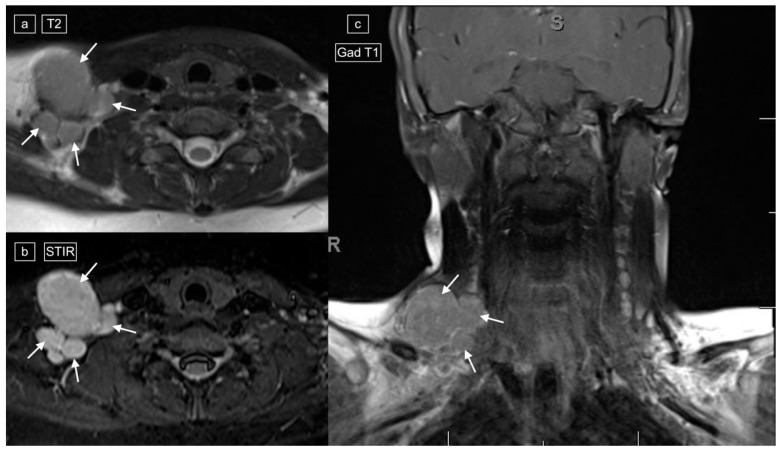
Images demonstrating technical reasons for lower T2 signal of lymphadenopathy. MRI of the cervical region in a 12-year-old male with a histologic diagnosis of nodular sclerosing Hodgkin lymphoma. The right lower cervical lymphadenopathy (arrows) demonstrates low signal on T2 (**a**) and higher signal on STIR (**b**), with only mild enhancement on post-gadolinium T1-weighted imaging (**c**).

This single-center, retrospective study was approved by the hospital’s Institutional Review Board and is compliant with the Health Insurance Portability and Accountability Act. The study was conducted at a large tertiary children’s hospital in the United States with advanced imaging services. MRI examinations were extracted retrospectively by using Illuminate (Softek Illuminate, Overland Park, KS, USA) with keyword searches on “MR” and “Hodgkin”. We further reviewed the medical records to include available chest and/or neck MR scans of patients with histologically confirmed nodular sclerosing Hodgkin lymphoma prior to initiation of chemotherapy. The MR studies were performed on a 1.5T or 3T MRI scanner (Siemens Healthineers, Malvern, PA, USA). An experienced pediatric radiologist (>20 years) reviewed the MR studies [Figure 1, Figure 2, Figure 3 and Figure 4] for lymph node signal on any available T2w and STIR sequences and recorded the presence of intermediate- or low-signal lymphadenopathy in any thoracic lymph node group. Other lymph nodes and lymphadenopathy, as well as normal soft tissue, were used as a benchmark for comparison. In addition, the T1 and post-gadolinium T1 sequences were evaluated for any central or peripheral enhancement. Results are presented as frequencies and percentages.

The study protocols were not standardized, as MR is not the standard investigation for lymphoma at our institution. Different protocols were employed with a variety of sequence parameters. Below is a composite of sequences and a variety of parameters for T2 and STIR [Table 1].

## 3. Results

We reviewed the chest and/or neck MR images of eleven children with biopsy-proven nodular sclerosing Hodgkin lymphoma. Median age at the time of MRI was 14.3 (IQR: 13.9–16.1) years, and nine were boys. The indications for the MR scans were as follows: a known diagnosis of Hodgkin lymphoma (*n* = 4), neck/chest masses (*n* = 4), MR exams referred from outside for a second interpretation (*n* = 2), and one case of chronic back pain with concern for chronic recurrent multifocal osteomyelitis. Of the eleven children, five showed some lymphadenopathy with low T2/STIR signal (45.5%), and six showed some lymphadenopathy with intermediate T2/STIR signal (54.5%). There were no children who demonstrated exclusively high-signal lymphadenopathy. Central gadolinium non-enhancement was observed in four patients (36.4%).

## 4. Discussion

This report focuses on the distinctive MRI signal characteristics of lymphadenopathy in histologically proven nodular sclerosing Hodgkin lymphoma. There are numerous articles in the literature that discuss the MRI features of lymphoma broadly [4,7,8,9]. Although there is literature that addresses the MRI features of Hodgkin lymphoma [1,10], there is a paucity of literature that has thoroughly correlated MRI signal characteristics with the biopsy-proven nodular sclerosis subtype, which is the predominant histological subtype of lymphoma in the pediatric population.

Diagnosis currently requires tissue sampling to characterize the lymphoma subtype according to the WHO classification. Imaging plays a key role in defining nodal involvement and identifying extra-nodal sites of disease. CT is the principal cross-sectional imaging modality, offering rapid assessment of the disease. It is usually coupled with 18 FDG-PET imaging to maximize the sensitivity of disease detection, especially for the assessment of bone marrow invasion. This allows accurate staging of lymphoma according to the Ann Arbor classification system [11,12].

Currently, MRI is utilized as the primary imaging modality only for the assessment of central nervous system and head and neck region lymphoma, where detailed anatomical evaluation is important [13,14]. However, MRI has been shown to have a similar sensitivity to CT in detecting nodal disease. Reports have shown that MRI has a 92% sensitivity in the detection of lymph nodes > 12 mm, as seen on CT, and can be a comparable, non-ionizing radiation imaging modality in the assessment of lymphoma [4,15,16]. Despite this, no clear guidelines have been published on implementing MRI protocols to be used for staging or diagnosing cases of lymphoma. This is particularly pertinent in assessing the pediatric population, as CT carries the risk of secondary radiation-induced lymphoma later in life, and MRI is therefore more desirable, especially in adolescents who would not require anesthesia or sedation for the MRI [17,18].

A variety of MRI/CT criteria have been suggested for identifying the involvement of lymph nodes, along with the criteria for standard size (longest diameter is greater than 1.5 cm) [19]. Lymph nodes may be deemed involved (i) if the diffusion-weighted imaging (DWI) signal surpasses that of the spinal cord, and (ii) the DWI signal remains elevated at higher b-values, showing restriction confirmed by a low apparent diffusion coefficient (ADC); (iii) in the presence of central necrosis, irrespective of nodal size; and (iv) when lymph nodes merge to form large nodal masses [20,21].

Regarding the evaluation of DWI quantitatively, despite the reproducibility of lymph node ADC measurements, there are currently no standardized cut-off values to distinguish normal lymph nodes from sites of lymphoma. Moreover, it remains undefined whether minimum or average ADC values should be employed for this goal [22]. Additionally, DWI/ADC exhibits several limitations in evaluating small thoracic lesions (hilar, mediastinal, and pulmonary) and those in tissues with inherently restricted diffusion patterns (e.g., the nervous system, renal parenchyma, and spleen). The former limitations stem from artifacts observed in DWI sequences due to cardiac pulsation and respiration, which may disrupt the ADC calculation. Furthermore, the challenging detection of lymphoma sites in organs with naturally low ADC values exacerbates this limitation under normal conditions [22,23].

T2 and STIR sequences can, therefore, present alternatives to DWI and ADC in assessing lymphoma. Previous studies have shown that T2/STIR sequences of all lymphatic tissue, including the thymus, usually exhibit high signal with great conspicuity of both benign and malignant lesions. To date, however, low T2/STIR signal has not been reported as a characteristic of lymphoma. However, one paper reports anecdotally that thymic lymphomatous involvement can demonstrate low-signal nodularity on fast spin echo STIR imaging, notably in a case of nodular sclerosing Hodgkin disease, but without linking the appearance to the histologic subtype [5]. The low and intermediate T2/STIR signal intensities of lymphadenopathy in our cases of biopsy-proven nodular sclerosing Hodgkin disease closely resemble those published in this paper.

A plausible explanation for the low/lower T2/STIR signal intensity observed in nodular sclerosis subtypes of Hodgkin lymphoma as seen in five of our patients who showed some lymphadenopathy with low T2/STIR signal [45.5%] and the six who showed some lymphadenopathy with intermediate T2/STIR signal [54.5%] may stem from the pronounced fibrosis seen with this histology. This fibrosis leads to reduced cellularity and, consequently, decreased water content within the tumors [24]. In contrast, other Hodgkin lymphoma variants which have high signal lymphadenopathy exhibit distinct cellular compositions: “lymphocyte-rich” types feature proliferating B immune cells, “lymphocyte-depleted” types display proliferation of cancerous Reed–Sternberg cells, and “mixed cellularity” types maintain a balance between these cell types [25,26].

Given these distinctions, it may seem reasonable to infer a correlation between the nodular sclerosis subtype and the hypointense/lower intensity T2 and STIR signals on MRI. Nevertheless, the scarcity of literature exploring the relationship between radiological and histological findings in lymphoma, coupled with the limitations of our small sample size, underscores the necessity for prospective studies on standardized magnets with standardized sequence parameters. Such investigations could shed light on the potential of these MRI sequences as biomarkers for nodular sclerosing Hodgkin lymphoma, which may obviate the need for invasive biopsies and inform patient management strategies.

We acknowledge some limitations in this study. This was a retrospective study with a relatively small sample size and without a control group. There was variability in scanning protocols and sequence parameters across different cases. Also, the study’s single-center design may limit the broader applicability of the results to other settings.

## 5. Conclusions

All eleven patients with a diagnosis of nodular sclerosing Hodgkin lymphoma showed some intermediate or low T2/STIR lymphadenopathy signal. Five children showed a frank low signal of some lymphadenopathy. To date, it has been reported that lymphoid tissue (including thymus) exhibits high T2/STIR signal with great conspicuity in both benign and malignant lesions, while low T2/STIR signal has not been reported as a characteristic of lymphoma. Based on these observations, it is reasonable to suspect a radio-pathological correlation between the nodular sclerosing subtype of lymphoma and T2 and STIR hypointense signal characteristics, which may prove to be a biomarker for this histology and warrants further research. We recommend prospective studies to explore this potential MRI biomarker of the nodular sclerosing subtype of Hodgkin lymphoma and its relevance to the management of patients, with a goal to avoid biopsy.

## Figures and Tables

**Table 1:** MRI sequence parameters.

	T2w	STIR
TR (repetition time) (msec)	4000–6000	4000–5200
TE (echo time) (msec)	100–140	35–50
Flip angle (deg)	140–160	140–160
Slice thickness (mm)	5	5
Matrix size	320 × 320	320 × 256
FOV (mm)	380	380
Parallel imaging	2	2
TI (inversion time) (msec)		190

## Data Availability

The data presented in this study are available on reasonable request from the corresponding author. The data are not publicly available due to privacy restrictions or ethical restrictions.
